# The Biomechanical Screening Game between Visitor Power and Staminode Operative Strength of *Delphinium caeruleum* (Ranunculaceae)

**DOI:** 10.3390/plants11172319

**Published:** 2022-09-05

**Authors:** Qin-Zheng Hou, Wen-Juan Shao, Nurbiye Ehmet, Guang Yang, Yu-Qin Zhong, Wen-Rui Min, Yi-Fan Xu, Ruo-Chun Gao

**Affiliations:** 1 College of Life Sciences, Northwest Normal University, Lanzhou 730070, China; 2 Physical Chemistry Biology Teaching and Research Group, Lanzhou Oriental School, Lanzhou 730070, China

**Keywords:** biomechanical, screening mechanism, *Delphinium caeruleum*, visitor power, operative strength, staminode

## Abstract

During the evolution of angiosperm flowers, some floral traits may undergo certain changes in order to participate in screening. The stamens and pistils of *Delphinium caeruleum* are covered by two “door-like” staminodes, the evolutionary function of which, however, is quite unknown. In this study, we investigated whether *D. caeruleum* staminodes acted as visitor filters by assessing the respective strengths of staminodes and visitor insects (six bee species). We measured the operative strength required to open the staminodes and the strength that insects were capable of exerting using a biological tension sensor. Furthermore, we compared the strength required to open staminodes at different phases of the flowering period (male and female phases) and the strength of different visitors (visitors and non-visitors of *D. caeruleum*). The results showed that the strength needed to open staminodes in the male phase was significantly higher than that in the female phase. There was no significant difference between the strength exerted by visitors and required by staminodes of *D. caeruleum* in the male phase, but the visitor strength was significantly higher than that required to open staminodes in the female phase flowers. The strength of non-visitors was significantly lower than that required to open staminodes in the male phase. Furthermore, there was a significant positive association between the strength and the body weight of the bees. These results highlighted the observation that only strong visitors could press the two staminodes to access the sex organs and achieve successful pollination. Furthermore, these results revealed the function of pollinator screening by the staminodes of *D. caeruleum*. The biomechanical approach to the study of flowers allowed us to address relevant ecological and evolutionary questions of the plant–pollinator interaction and explore the functional modules within the flower structure in other plant species.

## 1. Introduction

Most examples of cooperation between species involve the exchange of different types of rewards and services between the two partners. Plant–pollinator interactions are examples of cooperation, whereby plants can offer a variety of rewards (nectar, pollen, shelter, for example), and visitors can successfully transfer pollen from anthers to stigmas. However, both parts of the interaction are selected over time to utilize an appropriate partner to achieve the maximization of the benefits. As a result, research on plant–pollinator interactions has given rise to challenging topics with respect to partner choice [1]. Game theory offers two insights into this problem: the signaling game and the screening game, with plants and pollinators being able to choose to establish cooperative relationships with high-quality partners through these two mechanisms [2].

Previous studies have found that plants could limit the rewards to visitors by setting up barriers [3,4,5], whereby a visitor-screening mechanism in some plants was considered to be an effective mechanism to allow access by better partners [6,7,8,9]. Some flower structures become barriers by acting as filters for preventing entry by larger visitors so that only small visitors can access the flowers when they are receptive [4,10,11]. Flowers of some species have moveable parts that must be actively manipulated by insects to achieve pollination. For example, the effective force required to open flowers of *Cornus canadensis* (0.1–0.5 mN) favors large pollinators (such as bumblebees) that move rapidly between inflorescences; it effectively excludes smaller, less mobile visitors (ants, for example) [12]. A flower visitor-screening mechanism can be termed “forcible” when a flower structure has to be actively moved by a visitor in order to access the flower rewards and perform pollination [9]. Córdoba and Cocucci called the mechanical strength needed to open such a forcible floral mechanism the “operative strength” [9]. The plant can impose costs on potential visitors in the form of a “demanding environment”, preventing visitors from approaching the rewards or forcing them to invest more time and energy into harvesting the rewards. When the costs and rewards are balanced, only the high-quality visitors (pollinators) will be able to enter such a flower in a demanding environment [13]. The study of the strength required for the operation of movable parts allows us to clarify the relevant aspects of flower functional morphology. However, although there have been some previous studies on the biomechanical screening mechanism of plants for pollinators, the question of whether special flower structures can operate mechanical force screening for efficient insect visitors has been mainly based on the morphological description and subjective speculation [14]. There are also some supporting data from experimental studies, but not enough to form a theoretical system of visitor biomechanical screening mechanism [9,12].

**Figure 1:**
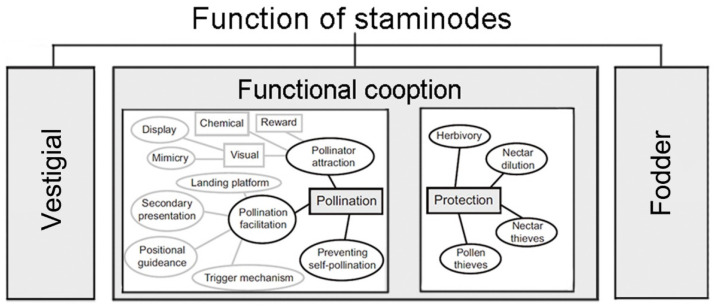
A schematic classification of staminodes based on evolutionary history and function. The staminodes are classified into three major groups based on their evolutionary history: vestigial, fodder, and functional cooption (modified from [19]).

During the evolution of flowers, stamens of some plants lost the main function of producing pollen and instead performed important secondary flower functions as staminodes. According to the “Most Effective Pollinator Principle” indicating that floral traits are shaped by the most frequent and effective local pollinators [15,16], the staminodes have been shown in some plants to have pollination-related functions [17,18], such as fodder, pollinator attraction, and protection (Figure 1) [19]. Recent studies have found that staminodes form physical barrier structures in some plant species and could play several different functional roles. The staminode of the three *Scrophularia* species (*S. lyrata*, *S. scorodonia*, and *S. canina*) flowers, for example, is found to protect the nectar from dilution by rainwater [20]. Such staminodes may also perform the function of insect screening. A study of *Penstemon digitalis* flowers indicated that the presence and function of the bristle staminodes were to affect size-dependent selection for bee body size and were associated with pollen-transport attributes [10]. In addition, some plants set up biomechanical barriers through the staminodes to limit the visitors from flower rewards (or increase the cost of rewards) to screen for effective pollinators [18]. This biomechanical screening function of the staminodes has not been reported before.

**Figure 2:**
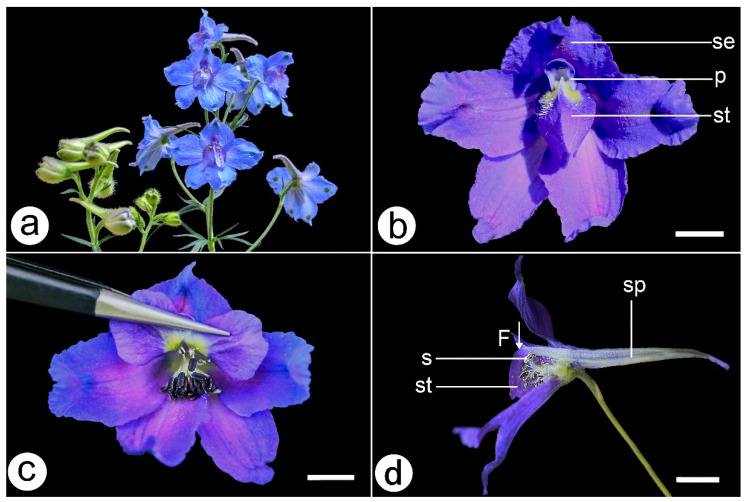
*D. caeruleum* in Gannan Tibetan Autonomous Region, China. (**a**), flowering plant of *D. caeruleum*. (**b**), detailed floral structure of *D. caeruleum* (p—petals, se—sepals, st—staminodes). (**c**), the pistils and stamens below the staminodes. (**d**), side longitudinal section view of *D. caeruleum* (s—stamens, sp—spur, st—staminode), arrow (F) denotes the direction in which visitors land and press the staminodes. Scale bars = 5 cm.

In this study, we selected *Delphinium caeruleum*, which is widely distributed on the Qinghai-Tibet Plateau, as the experimental subject. In the Ranunculales, the staminodes are diverse in position, morphology, abnormal development stage, and function [21,22]. A notable character of the genus *Delphinium* (Ranunculaceae) is the presence of two staminodes per flower, covering the anthers and stigmas [23,24] and forming a “double door” type of structure (Figure 2). Based on our preliminary observations, the visitors have to open this “double door” staminode structure to achieve complete pollination by making contact with the stamens and pistils below the staminodes. As a result, enough power needs to be generated by pollinators to promote cooperation between plants and pollinators. The staminodes of *Delphinium* species may require enough power to achieve opening to exclude ineffective visitors and to select effective partners. It had previously been suggested that the weight of the pollinators was insufficient to trigger such a mechanism, and additional muscular power exerted by the pollinators was needed [25]. Previous studies on the role of visitor strength in flower–pollinator interactions have been carried out on species belonging to different plant families (such as the Orchidaceae and the Cornaceae), or comparative studies have been performed among species within the same family (such as the Leguminosae) [9,12,26]. However, the function of the staminodes of *Delphinium* flowers has not been reported before. Furthermore, the selection of floral traits that increase attractiveness to pollinators is predicted to occur primarily through male function (pollen donation). Previous studies have suggested that although male success is not limited by pollinators, male-biased selection may still nevertheless occur if plants with traits such as large flower sizes have an advantage in donating pollen [27,28]. As complete protandry occurs in *D. caeruleum* [29], mating success due to pollen dispersal is more likely than pollen receipt to be limited by visitors [30], so we speculate that the operative strength of the staminodes may be different between the male phase and the female phase. However, no report has compared the operative strength of the same plant species of flowers at different developmental stages. We have tested the power generated by visitors, non-visitors, and staminodes of *D. caeruleum* and collected data separately on flower at the male phase and the female phase, aiming to test the following hypotheses: (1) the operative strength of the staminodes operates as a biomechanical visitor-screening mechanism by allowing visitors who are strong enough to open the staminodes of *D. caeruleum* to achieve pollination; (2) the operative strength of the staminodes of *D. caeruleum* in the male phase differs from that in the female phase; and (3) the strength exerted by visitors is related to their body weights and lengths.

## 2. Results

### 2.1. Floral Traits

**Figure 3:**
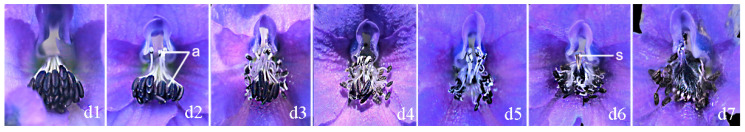
The flowering period of a single flower of *D. caeruleum* (a—anthers, s—stigmas). male phase of 1–5 d, female phase of 6–7 d.

Each flower of *D. caeruleum* lasted, on average, for 8.60 ± 1.51 d (n = 30). The flowers of *D. caeruleum* are protandrous, with the male phase lasting on average for 6.10 ± 0.99 d and the female phase lasting on average for 2.40 ± 1.45 d (Figure 3). During the flowering period of a single flower, the stamens occupied the center of the flower initially, then the stamens matured in sequence and moved. After that stage was completed, the styles elongated, and the stigmas occupied the central position and opened (Figure 3). This phenomenon indicated that both herkogamy (spatial separation of stamens and pistil in the same flower) and dichogamy (time separation of stamens and pistil open in the same flower) occurred in *D. caeruleum*.

### 2.2. Visitor Observations

**Figure 4:**
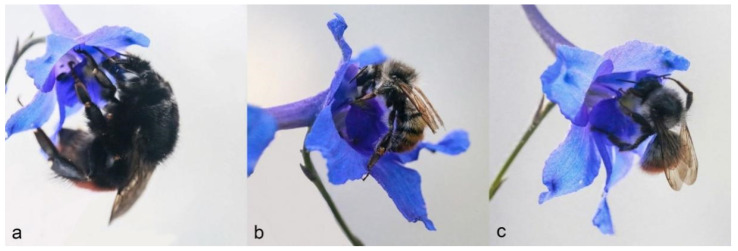
The visitors of *D. caeruleum* in Gannan. (**a**) *B. pyrosoma*. (**b**) *B. impetuosus*. (**c**) *B. lepidus*.

**Figure 5:**
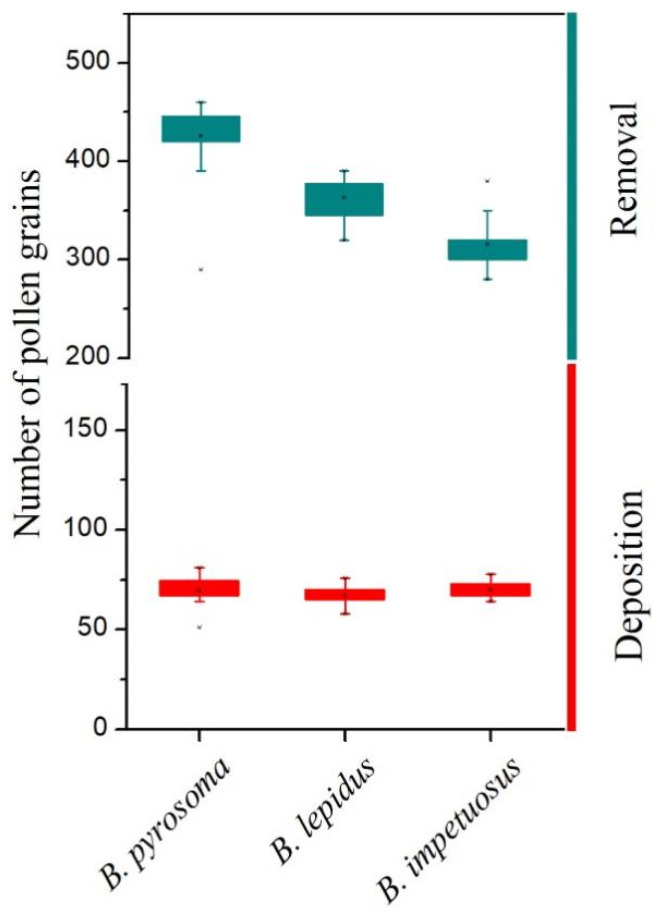
Mean (± SD) amount of pollen grains deposited on stigmas and pollen removed of visitors on the *D. caeruleum*. Boxplots represent the number of pollen grains deposition and amount of pollen removed (red for pollen deposition and dark cyan for pollen removed by visitors), showing medians, quartiles, interquartile ranges, and outliers.

Because we did not observe insects visiting flowers at night, the diurnal times at which visitors were observed were recorded and analyzed. We observed that the local visitors to flowers of main plant species in the station area were the bumblebees, including *Bombus lepidus*, *B. pyrosoma*, *B. impetuosus*, *B. kashmirensis*, and *B. sichelii*, and the honeybees, including *Apis mellifera*. Among these insects, three bumblebees, *B. lepidus*, *B. pyrosoma*, and *B. impetuosus*, were regular visitors to *D. caeruleum* (Figure 4), whereas the other three species did not visit the flowers of *D. caeruleum*. We observed that all the individuals of each visitor tested behaved in the same way when visiting flowers. First, the visitor (all were bumblebees) landed on the staminodes with their fore and middle legs clasped onto the staminodes and their hind legs on the lower sepals. After this, the visitors pushed their proboscis (in most cases accompanied by the head) between the two petals by vertically pressing downward on the staminodes. Artificial simulation experiments indicated that the bees had to press vertically downward on the staminodes to contact the anthers (Figure 2). The “double door” staminodes opened under the downward pressure of insects, and the sex organs hidden beneath were presented (Figure 4). Based on the behavior and efficient pollination rate of these three visitors, we conclude all are pollinators of *D. caeruleum* (Figure 4 and Figure 5).

All visitors of *D. caeruleum* were efficient at pollen deposition and removal. The mean ± SD number of pollen grains removed by visitors on the *D. caeruleum* were 426.4 ± 32.38 (*B. pyrosoma*), 360.87 ± 20.79 (*B. lepidus*), and 316.57 ± 21.75 (*B. impetuosus*), respectively (n = 30). The mean ± SD number of pollen grains deposited on stigmas by visitors to *D. caeruleum* were 69.57 ± 5.55 (*B. pyrosoma*), 67.30 ± 4.56 (*B. lepidus*), and 69.80 ± 3.80 (*B. impetuosus*), which have no significant differences (Figure 5). We, therefore, conclude that one or two effective visits by any of the three species visitors may ensure large-scale ovule fertilization of *D. caeruleum*.

Field observations in the study area indicated that the number of visit times by the three pollinators was relatively low. When the staminodes were removed, the pollinator composition did not change, but the visit times of the three pollinators were significantly lower than natural (Table 1). Additionally, the number of visit times of the three pollinators in the male phase were much higher than those in the female phase (Table 1).

### 2.3. Relationships between Insect Strength and Both Body Weight and Length

**Figure 6:**
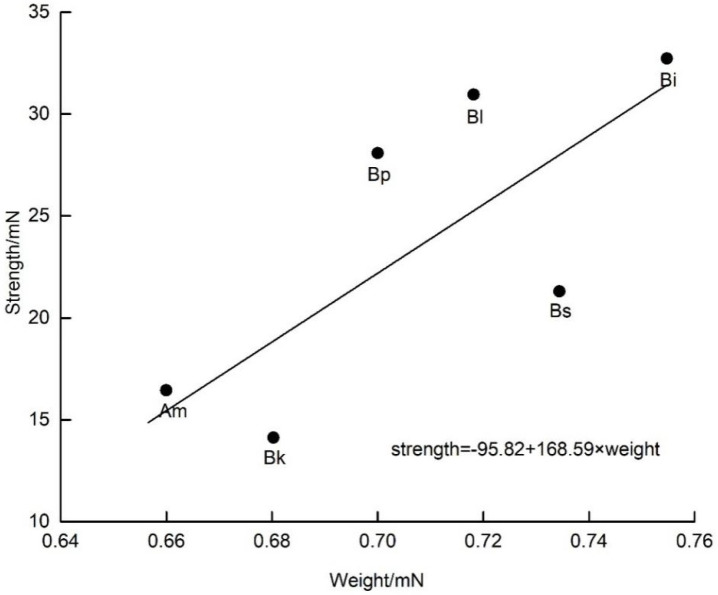
Linear regression with visitors’ weight as an independent variable and strength as a dependent variable. *p* = 0.022; r^2^ = 0.613. Am, *A. mellifera*. Bi, *B. impetuosus*. Bk, *B. kashmirensis*. Bl, *B. lepidus*. Bp, *B. pyrosoma*. Bs, *B. sichelii*.

**Figure 7:**
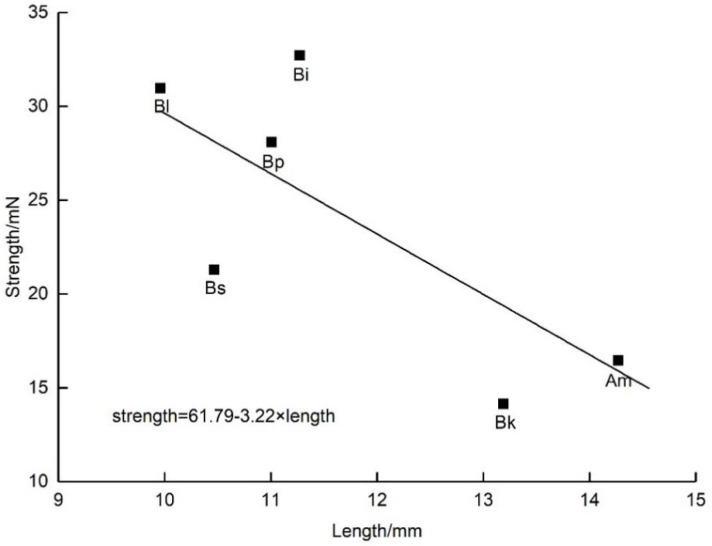
Linear regression with visitors’ length as an independent variable and strength as a dependent variable. *p* = 0.016; r^2^ = 0.651. Am, *A. mellifera*. Bi, *B. impetuosus*. Bk, *B. kashmirensis*. Bl, *B. lepidus*. Bp, *B. pyrosoma*. Bs, *B. sichelii*.

The mean ± SD weight and the average body lengths were significantly different among the six native insect species in the study area (Table 2). The strengths generated were also significantly different among these insect species (Table 2). Correlation analysis showed that the strengths generated by the different bee species (including both visitors and non-visitors of *D. caeruleum*) were significantly positively correlated with insect weight (r = 0.783, *p* < 0.05; Figure 6) but were significantly negatively correlated with body length (r = −0.807, *p* < 0.05; Figure 7), suggesting that stocky (short but heavy) bee species were strong enough to act as pollinators.

### 2.4. Flower Operative Strength and Visitor Strength

**Figure 8:**
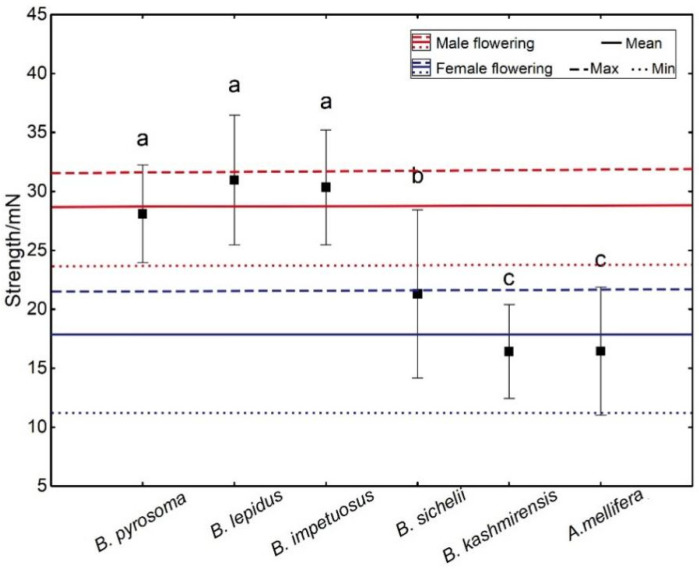
Operative strength of *D. caeruleum* and local visitors. *B. impetuosus*, *B. lepidus*, and *B. pyrosoma* are visitors of *D. caeruleum*. Means ± SD are shown. The bars with different letters (a, b, and c) indicate significant differences between the means at *p* < 0.05.

The operative strength of the staminodes in male phase flowers (29.02 ± 2.86 mN) was significantly higher (*p* < 0.05) than the operative strength in female phase flowers (17.81 ± 2.55 mN). The strengths generated by the visitors (30.34 ± 4.86 mN, 30.96 ± 5.51 mN, and 28.09 ± 4.16 mN for *B. impetuosus*, *B. lepidus*, and *B. pyrosoma*, respectively) were not significantly different from the staminode operative strength in flowers at the male phase but were significantly higher than those in female phase flowers (*p* < 0.05; Figure 8). Non-visitors of *D. caeruleum* generated significantly lower strengths than those of visitors (*p* < 0.05), with the strengths of the non-visitors being significantly lower than the operative strength of the staminodes in male phase flowers (*p* < 0.05; Figure 8).

## 3. Discussion

Staminodes are common floral structures that help both to attract visitors and to protect the sex organs [20,29], but our observations found that visitors of *D. caeruleum* contacted the anthers and stigmatic surface by opening the door-like structure of the staminodes. A considerable number of plant species are known, e.g., members of genera such as *Salvia*, *Cornus*, *Calceolaria*, etc., where flowers have movable parts, which are obstacles that must be actively moved by a visitor to access the flower rewards and achieve pollination [12,26]. In our study, we observed that only strong visitors pollinated *D. caeruleum*. Consequently, we speculated that weak bees were unable to trip open the staminodes, which were stronger than themselves because we did not observe weak bees visiting flowers. The strength exerted by insects was clearly related to their body size, and the shorter but heavier ones generated larger strength (Figure 6 and Figure 7). Compared with other local visitors (non-visitors of *D. caeruleum*), the three pollinators of *D. caeruleum* were all strong bumblebees, which were able to overcome the mechanical barriers imposed by the staminodes in both the female and male phases of flowering, whereas the weak non-visitor insects were unable to contact the anthers and stigmas because they could not exert sufficient downward pressure on the staminodes. Bumblebees are more efficient pollinators [31], which was confirmed by the high pollen deposition and removal efficiency of pollinators (Figure 5). Therefore, one or two effective visits by bumblebees may ensure large-scale ovule fertilization and subsequent seed set of *D. caeruleum*. So, *D. caeruleum* may separate high-quality pollinators from other visitors by this screening mechanism, although in the study, it was difficult to measure the pollen deposition and removal efficiency of non-visitors. Previous studies found that bumblebees were more efficient than honeybees in pollination parameters, such as floral visitation rates and stigmatic pollen deposition, possibly, as strategies for growing larvae early in the season and for queen development in late summer, individual bumblebees make more foraging trips in a day and bring back more pollen to the hive each time, compared with honeybees [32,33]. Therefore, we considered the presence of staminodes selected for visits to *D. caeruleum* by only high-quality visitors, with the staminodes acting as mechanical visitor screens. This function of the staminodes also solves the problem of partner selection in the interactions between visitors and flowers of *D. caeruleum*.

To our knowledge, our study is also the first to explore whether the operative strength of flowers varied between the male phase and the female phase of flowering. This is an important phenomenon because hermaphroditic flowers have the potential to allow the evolution of differences in floral traits in the male and female phase flowers [34]. Plants prefer to interact with high-quality visitors (such as those exhibiting high pollination efficiency). All the bees examined in a previous study on visitor strength exhibited strengths greater and frequently several times greater than were needed to open the floral mechanism of the species they visited [9]. We speculate that these differences between the operative strength of staminodes and the relative strength of the insect at the male and female phases in *D. caeruleum* were probably the results of the functional evolution of the staminodes. Previous studies have found that the evolution of attractive traits of flowers is assumed to occur primarily through selection for male fitness [34] because male mating success is more likely to be limited by the amount of pollen dispersed by visitors, whereas female fitness may be maximized by just a few visitor visits that bring adequate pollen amounts to achieve full seed set [35]. Staminodes can select “effective” visitors by operating a biomechanical screening function to ensure that visitors can transfer pollen in the male phase, and this function does not exist during the female phase because all the strong visitors selected by the flower in the male phase are able to negotiate the staminodes in order to deposit pollen onto stigmas in the female phase, whereas weak bees are unable to transfer pollen to achieve complete pollination in the male phase. Hence, the staminodes of *D. caeruleum* formed a “biomechanical visitor-screening mechanism” only in the male phase, a barrier that prevented non-visitors from approaching the rewards or which forced cooperative visitors to invest more time and energy in harvesting rewards and transferring pollen.

By imposing a strategic cost on visitors, even though the plant cannot directly assess the quality of potential visitors, the visitors will screen themselves according to their own quality [13]. Consequently, *D. caeruleum* flowers can distinguish high-quality visitors from other potential visitors. In order to make this strategy possible, the strengths of the visitors must be great enough to open the staminodes. Biomechanical traits, such as closed corollas in *Phlomis* [14] and pollen catapults in *Cornus canadensis* [12], could confront visitors with physical obstacles that only high-quality visitors are able to overcome. Even when all visitors can overcome the biomechanical barriers, such as staminal levers in *Salvia* [26] and papilionate legume flowers [9], these structures can still be examples of a demanding environment if they impose energy or time costs on the visitors. We concluded that, by properly setting up such a demanding environment, the plants could ensure visits by only high-quality visitors.

## 4. Materials and Methods

### 4.1. Study Site

The study was performed at the Gannan Grassland Ecosystem Field Science Observation and Research Station of the Ministry of Education, Alpine Meadow and Wetland Ecosystem Positioning Research Station of Lanzhou University (34°55′ N, 102°53′ E; altitude 3300 m), in HeZuo City, China. The station is located on the eastern edge of the Qinghai-Tibet Plateau, with an annual mean temperature of 2.4 °C and annual mean precipitation of 530 mm, the latter occurring mostly from July to August.

### 4.2. Plant Species

*Delphinium caeruleum* is an entomophilous, perennial herb, which grows in alpine meadows, at altitudes of 2500–4200 m. According to Cruden’s [30] dividing standards, the mating system of *D. caeruleum* belongs to obligate xenogamy (unable to achieve autonomous self-pollination) [30]. The flowering period of *D. caeruleum* is from July to September. Each flower on the corymbose *D. caeruleum* inflorescence contains five purple-blue sepals and two petals, which end with an extension to form a nectar spur within the sepal spur. Two blue staminodes with yellow barbate are located in the center of the flower, and the stamens and pistils are at the bottom of the two staminodes [24] (Figure 2).

### 4.3. Floral Traits

To determine the pollen and stigma presentation in flowers, 15 fresh flowers on 15 individual plants (i.e., one bud per plant) of *D. caeruleum* were randomly selected to be observed every four hours from 8:00 a.m. to 8:00 p.m. in situ during the flowering period without picking flowers. The pollen presentation phase (male phase) was recorded as the time between the first stamen beginning to lift up and the last stamen falling back. The stigma presentation phase (female phase) was recorded as the time from the initial stigma lobes opening to the sepals wilting.

### 4.4. Visitor/Non-Visitor Observations

At the peak of the flowering period, we randomly selected 30 individual plants (the flowers of 15 individual plants were with removed staminodes) to conduct visitor observations at the same time on five sunny days. We recorded whether each flower was in the male phase or in the female phase every day. During the surveys, we observed and recorded the number of visit times of the different treatments (natural, removed staminodes; in the male phase, in the female phase) of flowers and the foraging behavior of visitor insects. To avoid interrupting the normal activity of the visitors, the observer stood quietly 1.5 m away from the plants to monitor the visitors during the daytime (from 8:00 a.m. to 20:00 p.m.), and we observed visitors at night with an infrared camera (from 20:00 p.m. to 8:00 a.m.). At the same time as visitor observation, we conducted observations on other insects (non-visitors of *D. caeruleum*). By monitoring visitors of co-flowering species of *D. caeruleum*, such as *Leontopodium* sp., *Potentilla* sp., and *Aster* sp., we recorded all the visitors to the station area.

Individuals of the insects in the station area (including visitors and non-visitors of *D. caeruleum*) were caught and deposited specimens with their specimen numbers in a specimen box for identification by entomologists. Two expert entomologists measured the anatomical traits of the bees to identify the species by a stereomicroscope; some species, which could not be identified solely on the grounds of morphology, were identified by a molecular method, and PCR sequencing, using cytochrome c oxidase I (COI) DNA barcodes, was performed and the sequences referenced to the BOLD database for identification [36].

To detect the pollen removal and pollen deposition of each visitor, we bagged 30 flowers on 30 inflorescences before the anthesis. All anthers and stigmas of the individual flower were carefully collected into separate centrifuge tubes after a single visit, and the visitor was captured for identification. The pollen deposition was measured by microscopic examinations after staining with lactophenol cotton blue. The pollen removal by visitors was recorded as the difference between the average number of pollen grains before dispersal and the number of pollen grains remaining after being visited.

### 4.5. Biomechanical Visitor-Screening Mechanism

**Figure 9:**
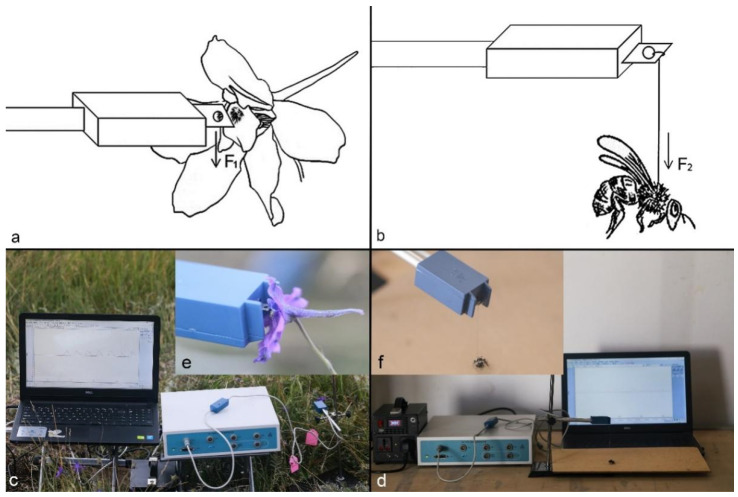
The strength of the staminodes ((**a**). F_1_) and the visitors ((**b**). F_2_) of *D. caeruleum* was measured by biological tension sensor. (**a**), schematic showing how the sensor was used to measure strength of a flower. (**b**), schematic showing how the sensor was used to measure strength of a bee. (**c**), the apparatus constructed to measure operative strength in flowers. (**d**), the apparatus constructed to measure operative strength in bees. (**e**), detail showing the flowers are handled to measure operative strength. (**f**), detail showing how the sensor is constructed to measure the strength exerted by visitors.

We found six species of bees (including visitors and non-visitors of *D. caeruleum*) frequently in the station area, and we caught the insects (n ≥ 20 per species) to carry out morphometric measurements. A biological tension sensor, capable of measuring from 0.001 g to 5 g (BL-420s Biological Function Experimental System and FT-102 Biological Tension Sensor; Techman Soft, Chengdu, China), was used to measure the strength of the visitors (Figure 9). When each visitor insect individual was suspended vertically on the metal plate of the sensor by a string attached to the chest of this live insect (diameter = 0.074 mm) and had just landed on a horizontal board, the completely downward straight pulling strength of the visitor on the metal plate of the sensor by the string, which was considered as the greatest strength of the visitors, was measured, because insects tried their most to escape at this time. In this way, we can verify whether insects can open staminodes. We measured the strength of each visitor species on 15 individuals. Insect weights were measured on an analytical balance, and the body lengths were measured with a Vernier caliper. A specimen number was assigned to each insect when we measured their strength, weights, and body lengths. We deposited specimens with their number in the specimen box for identification by entomologists.

Through field observations, 30 fresh flower buds on 30 individual plants (i.e., one bud per plant) were randomly selected to measure the operative strength necessary to press the staminodes of attached *D. caeruleum* buds in situ during the flowering period without picking flowers. We measured the operative strength of staminodes by artificially pressing the staminodes with the metal plate of the FT-102 Biological Tension Sensor until the stamens were exposed (with three measurements on each flower) (Figure 9). We measured the in situ operative strength of individual attached flowers, with 15 measurements being carried out on flowers in the male phase and 15 on flowers in the female phase. The strength of visitors and staminodes was expressed in mN.

### 4.6. Data Analysis

All experimental results are presented as the mean ± standard deviation (SD). The single sample Kolmogorov–Smirnov non-parametric normality test was used to check whether the test data followed the normal distribution. The strength of pollinators and the operative strength of staminodes both exhibit a normal distribution (*p* > 0.05), and thus we used a one-way analysis of variance (ANOVA) to analyze the differences. We used a paired sample t-test to compare the operative strength of staminodes at the male and female phase. The normality of pollen removal and pollen deposition was tested by 1-K-S, and we used one-way ANOVA for variables exhibiting a normal distribution. Statistical analyses were performed using SPSS 23.0 (IBM, Armonk, NY, USA). To study the relationship between the body weight or length with the strength of the visitors (using the mean value from each species for each variable), linear regression and correlation analysis was carried out with the strength of visitors as the response variable and weight or length of visitors as the independent variable, respectively, using Origin 9.1 software (OriginLab, Northampton, MA, USA).

## 5. Conclusions

The ecological value of staminodes in flowers has rarely been studied. Previous studies have shown that the function of the staminodes of *D. caeruleum* was to attract visitors and to protect the pistils and stamens, with its biomechanical visitor-screening mechanism being almost completely overlooked. Our study suggested a new functional perspective on staminodes: screening of efficient pollinators of *D. caeruleum* was achieved through a biomechanical screening mechanism base on the staminodes, with benefits for both the plant and the visitors. It indicated that there was a biomechanical visitor-screening mechanism based on the operative strength of staminodes, which allowed access to nectar and pollen only to visitors strong enough to press the staminodes of *D. caeruleum* flowers with sufficient downward pressure to access and transfer the pollen. Non-pollinators do not avoid flowers simply because of the influence of flower scent, color, and other factors [37,38]. Significant strength is necessary for insects to successfully collect the reward by manipulating the staminodes of *D. caeruleum*, which act as a threshold to be overcome by effective pollinators.

The biomechanical approach to the study of flower biology allowed us to address relevant ecological and evolutionary questions of a newly discovered and important function of staminodes. More importantly, it can address the evolutionary and ecological questions of relevant biomechanical visitor-screening mechanical in plant–pollinator interactions and provides the possibility for further exploration of functional modules in floral structures in other species. Only descriptive studies have been conducted on the biomechanical screening mechanisms for floral traits in a few species of Orchidaceae, Cornaceae, Ranunculaceae, and Fabaceae. This mechanism may also exist in many plant species, which has not been discovered, not to mention in-depth research, which is insufficient to form a theoretical system of insect screening mechanism. In addition, there may be other ways for plants to screen for effective insects that have not yet been discovered and need to be further explored in the future.

## Figures and Tables

**Table 1:** The number of visit times by visitors to flowers of *D. caeruleum* with different treatments in different flowering phases.

Species	Male Phase		Female Phase		Total
	Intact	Staminodes Removed	Intact	Staminodes Removed	
*B. impetuosus*	18	2	8	1	29
*B. lepidus*	10	9	2	1	22
*B. pyrosoma*	12	4	1	2	19
Total	40	15	11	4	70

**Table 2:** The weight, length, and strength of visitors. *B. impetuosus*, *B. lepidus*, and *B. pyrosoma* are visitors of *D. caeruleum*. Means ± SD are shown. Different letters (a, b, and c) within the same column indicate significant difference at *p* < 0.05.

Species	N	Weight/g	Length/mm	Strength/mN
*A. mellifera*	62	0.66 ± 0.09 b	14.27 ± 1.56 a	16.45 ± 5.43 c
*B. impetuosus*	39	1.02 ± 0.46 a	11.27 ± 1.29 b	30.34 ± 4.86 a
*B. kashmirensis*	23	0.68 ± 0.13 b	13.19 ± 1.60 a	16.41 ± 3.98 c
*B. lepidus*	23	0.72 ± 0.33 b	9.96 ± 1.85 c	30.96 ± 5.51 a
*B. pyrosoma*	26	0.70 ± 0.14 b	11.01 ± 0.91 b	28.09 ± 4.16 a
*B. sichelii*	48	0.73 ± 0.44 b	10.47 ± 1.26 bc	21.31 ± 7.13 b

## Data Availability

All of the data provided in this study are available within this article.
